# Hepatic artery stenting with Viabahn

**DOI:** 10.1186/s42155-024-00507-w

**Published:** 2024-12-19

**Authors:** Kenichiro Okumura, Takahiro Ogi, Junichi Matsumoto, Nobuyuki Asato, Xiamin Sun, Hirohito Osanai, Kazuto Kozaka, Satoshi Kobayashi

**Affiliations:** Department of Radiology, Kanazawa University Graduate School of Medical Sciences, 13-1 Takara-Machi, Kanazawa, 920-8641 Japan

**Keywords:** Hepatopancreatobiliary bleeding, Endovascular treatment, Viabahn stent grafts, Vessel morphology, Stent patency

## Abstract

**Background:**

The effect of vessel morphology on the technical success and patency of Viabahn stent-grafts in treating postoperative arterial injuries and bleeding (AIB) after hepatopancreatobiliary surgery is not well understood. Difficulties in stent insertion persist despite using stiff guidewires to straighten tortuous vessels. This study aimed to identify vessel morphologies linked to technical success and short-term patency and to explore effective management strategies.

**Materials and methods:**

This retrospective study examined 12 consecutive cases of hepatic artery stenting in 11 patients, using Viabahn grafts for postoperative AIB from 2017 to 2024. Patient data, angiographic outcomes, and stent placement details were reviewed. Different types of guidewires, including stiff and soft guidewires, were utilized to facilitate stent deployment. Vessel tortuosity and vessel narrowing before stent placement were evaluated both qualitatively and quantitatively. Outcomes measured included technical and clinical success rates, stent patency at one month, and the time from surgery to stent placement.

**Results:**

Final technical and clinical success was achieved in all cases (100%). Vessel tortuosity often led to the emergence of accordion-like appearances upon vessel straightening, necessitating additional technical adaptations due to the formation of steps (*p* = 0.005). One-month stent patency was observed in 10/12 cases (83%). Among cases with severe vessel narrowing distal to the bleeding point, 2/3 (67%) experienced stent occlusion, significantly higher than those with less severe narrowing (*p* = 0.045). All occluded cases involved the extension of stent length by overlapping stent-grafts.

**Conclusions:**

Steps created by the accordion-like appearance in the hepatic artery resulting from the straightening of tortuous vessels can complicate stent insertion, and severe narrowing distal to the bleeding point increases the risk of short-term occlusion.

## Introduction

Arterial injuries, such as pseudoaneurysms, extravasation, and bleeding (AIB) that occur after abdominal surgeries are rare but pose significant risks with mortality rates between 10–40% [1, 2]. Traditionally, these injuries have been treated surgically, but surgical management can be challenging due to extensive tissue damage and inflammation following dissection, especially near arterial stumps [3]. Consequently, minimally invasive endovascular treatments, particularly transarterial embolization using embolic materials, have become the preferred approach [4–13]. The incidence of ischemia during hepatic artery embolization ranges from 29–70% [14, 15] and could sometimes lead to fatal liver failure [16], necessitating careful consideration of the indications for this procedure. Stent grafts, especially self-expanding Viabahn stent-grafts, are increasingly favored for their dual ability to achieve hemostasis while maintaining blood flow to peripheral organs [17, 18]. In Japan, the Viabahn stent graft is approved and covered by insurance for treating vascular injuries, including arterial injuries. There is growing interest in both the short- and long-term outcomes of its use for hepatic artery injuries [19–26]. Nevertheless, the variability in reported success rates, the rarity of these cases, and the insufficient understanding of factors influencing technical success may cause hesitation in performing the procedure. We hypothesized that certain aspects of hepatic artery morphology might critically affect the outcomes. The accordion phenomenon, which occurs due to mechanical distortion of a straightened vessel during coronary and vascular interventions, has been noted in the hepatic artery [27]; however, comprehensive reports on this subject are limited, and how it may impact stent insertion remains unclear. Moreover, placing a stent graft in the hepatic artery that is less than 4 mm in diameter or oversizing the stent graft by more than 20% are known risk factors for occlusion [22]. Furthermore, during stent placement, the patient's unstable condition and variable vessel appearance make accurate assessment of the vessel diameter challenging.

This study aimed to clarify the relationship between vessel morphology observed on digital subtraction angiography and the successful placement and short-term occlusion of the Viabahn stent graft in treating postoperative AIB following hepatopancreatobiliary surgery.

## Materials and methods

### Study design and patients

This retrospective study included all consecutive patients who presented with hepatic artery bleeding between January 2017 and May 2024 at our institution. The study was conducted in accordance with the CONSORT guidelines for reporting clinical trials [28].

### Patient selection

**Fig. 1:**
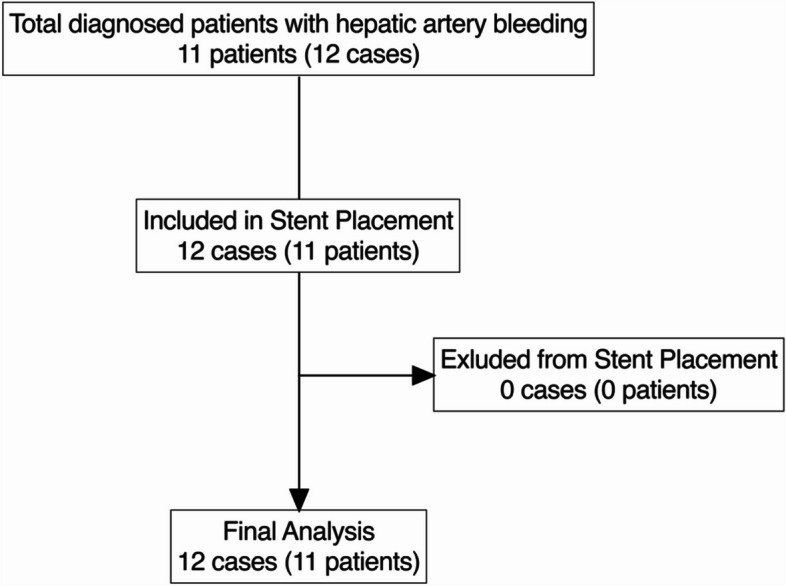
Flow diagram illustrating the patient selection process for emergency endovascular treatment of hepatic artery bleeding. All 11 patients (12 cases) diagnosed with hepatic artery bleeding underwent Viabahn stent graft placement, resulting in 12 cases (11 patients) included in the final analysis. No patients were excluded from stent treatment

During the study period, 11 patients with 12 cases of hepatic artery bleeding were diagnosed based on computed tomography (CT) angiography showing pseudoaneurysms or extravasation after surgery. All cases received emergency endovascular treatment using Viabahn stent grafts and were included in this study (Fig. 1).

### Data collection

Demographic and clinical data were collected for all patients, including sex, age, primary disease, surgical procedure, bleeding site, diameter of vessels and stent-grafts, stent graft placement length, overlap length, technical success of stent placement, clinical success (including bleeding-related death and liver failure), stent patency at 1-month post-operation, and any recorded complications and deaths.

### Endovascular procedure

Endovascular procedures were performed via the common femoral artery under local anesthesia. A 0.035 or 0.032-inch guidewire (Radifocus Guide Wire M; Terumo, Tokyo, Japan) was used to insert a 6–7-Fr guiding sheath (Destination; Terumo, Tokyo, Japan). A 4.2-Fr catheter (STEXDJ; Hanako Medical, Tokyo, Japan) was advanced to selectively target the celiac artery. A 2.4-Fr microcatheter (Progreat; Terumo, Tokyo, Japan) was then advanced over a 0.016-inch microguidewire (Meister; Asahi Intecc, Aichi, Japan) was used to advance the microcatheter to the distal side of the injured artery. The floppy tip of the soft guidewire was advanced into the intrahepatic circulation, while the stiffer portion was positioned across the arterial injury to straighten and support the injured segment. No anticoagulation with heparin was administered during the procedures. The guidewire was switched to a 0.018-inch wire (V-18TM, Boston Scientific, Natick, MA, USA; VASSALO GT, Cordis, Miami, USA; Treasure floppy, ASAHI INTECC, Aichi, Japan; Aguru, Boston Scientific, Natick, MA, USA), for stent graft delivery. Guidewires were stratified based on the perceived stiffness of the shaft. ASAHI Treasure floppy was considered soft, V-18™ wire intermediate, and VASSALO GT wire stiff. This classification was determined by tactile differences experienced during use. Initially, the V-18TM wire was used for all procedures as recommended by the manufacturer. In emergencies when the V-18TM was unavailable, a similar guidewire readily available was used. If resistance was encountered while advancing the stent graft over the guidewire, we exchanged the wire for a stiffer one to facilitate delivery. The guiding sheath was placed as close as possible to the bleeding point, extending beyond the celiac artery, and the stent graft was advanced over the bleeding point without further advancing the sheath. The injured artery was sized using arteriography or preoperative CT angiography. The stent graft diameter was approximately 110% of the injured artery diameter, and its length covered the entire injury with at least a 1 cm landing zone proximally and distally, facilitating secure placement and addressing the distal vascular stenosis beyond the bleeding site that this was possibly due to compression of the artery. If the proximal vessel diameter was large, the size was adjusted accordingly. Since the minimum available stent diameter was 5 mm, placing it in vessels smaller than 4.5 mm meant exceeding the recommended practice of oversizing the stent graft by approximately 10%. If a type 1 endoleak was identified, further percutaneous transluminal angioplasty was performed a PTA micro-balloon catheter (Rx-Genity; Kaneka Medix, Osaka, Japan) with a larger or same stent graft diameter. A Viabahn stent graft (Gore, Flagstaff, AZ, USA) with diameters ranging from 5 to 13 mm and lengths of 25 or 50 mm was deployed to cover the entire injury site. If a branch artery was within 5 mm of the injured site, it was embolized using coils (Tornade, Cook Medical, Bloomington, IN, USA; Interlock, Boston Scientific, Natick, MA, USA) to prevent a type 2 endoleak. Angiography was performed immediately after stent graft deployment. Post-dilatation was not performed if the stent graft was adequately expanded. Following the procedure, unless contraindicated, patients were prescribed dual antiplatelet therapy with aspirin (100 mg/day) and clopidogrel (75 mg/day) for six months to prevent stent graft thrombosis. However, adjustments to the duration and dosage were made based on the bleeding risk profile of each patient. In addition, systemic heparinization and the reduction of high-dose antiplatelet agents were employed as needed to effectively manage patient care. Follow-up CT angiography was performed one month post-treatment to monitor the stent grafts and assess vascular integrity. The imaging protocol consisted of dynamic CT scans capturing plain, arterial, and venous phases. Technical success was defined by the successful deployment of the stent, cessation of bleeding, and preservation of hepatic arterial blood flow. Clinical success was defined as the absence of mortality due to liver failure.

### Evaluation criteria

#### Qualitative classification of accordion-like formations after straightening

**Fig. 2:**
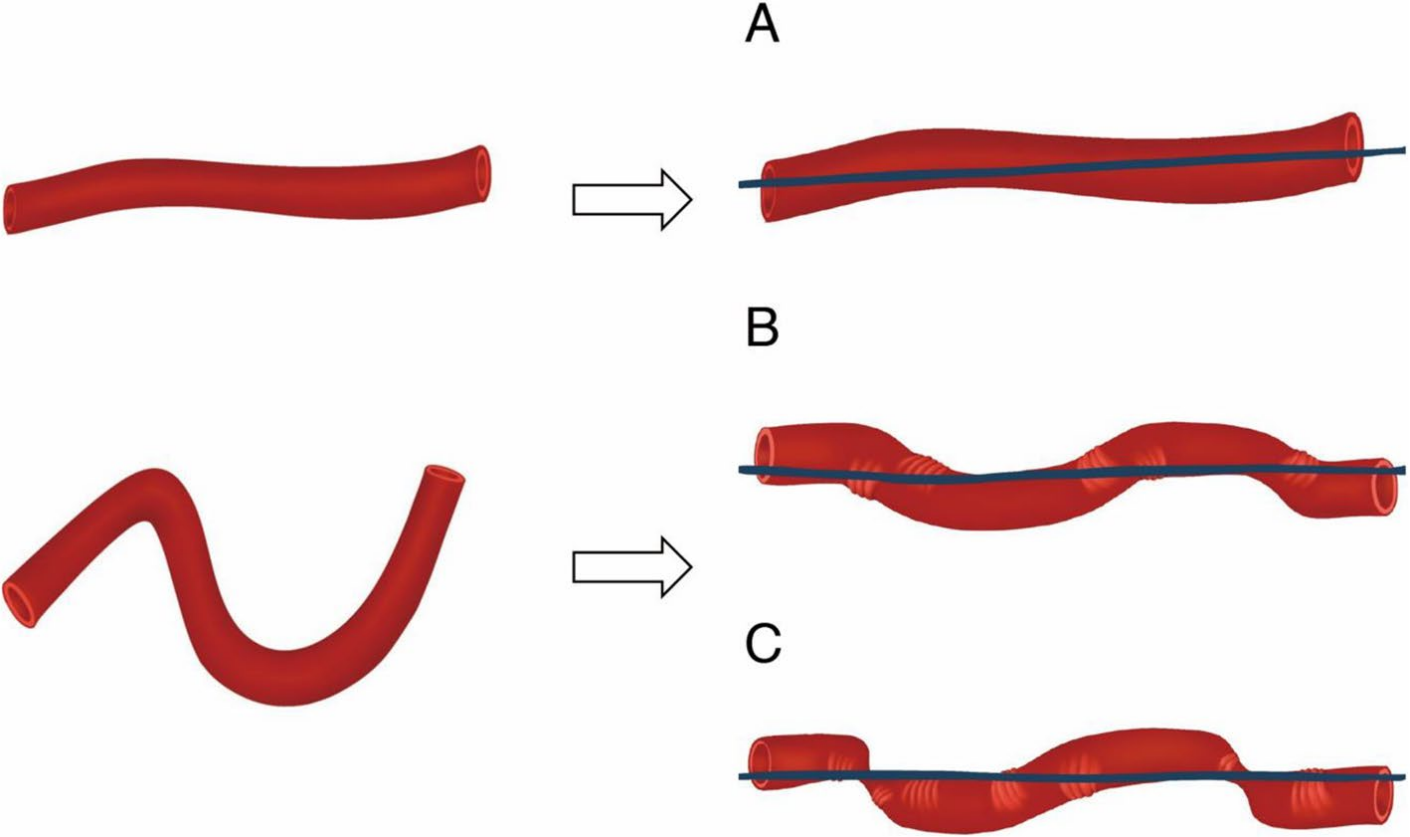
Angiographic Findings During Vessel Straightening. This schema illustrates the angiographic changes observed during vessel straightening using a guidewire. **A** The left side shows the original, unstraightened vessel configuration. **B** The vessel exhibits an accordion-like appearance due to compression. **C** The vessel shows both accordion-like patterns and step formation, indicating multiple areas of compression and extension

**Fig. 3:**
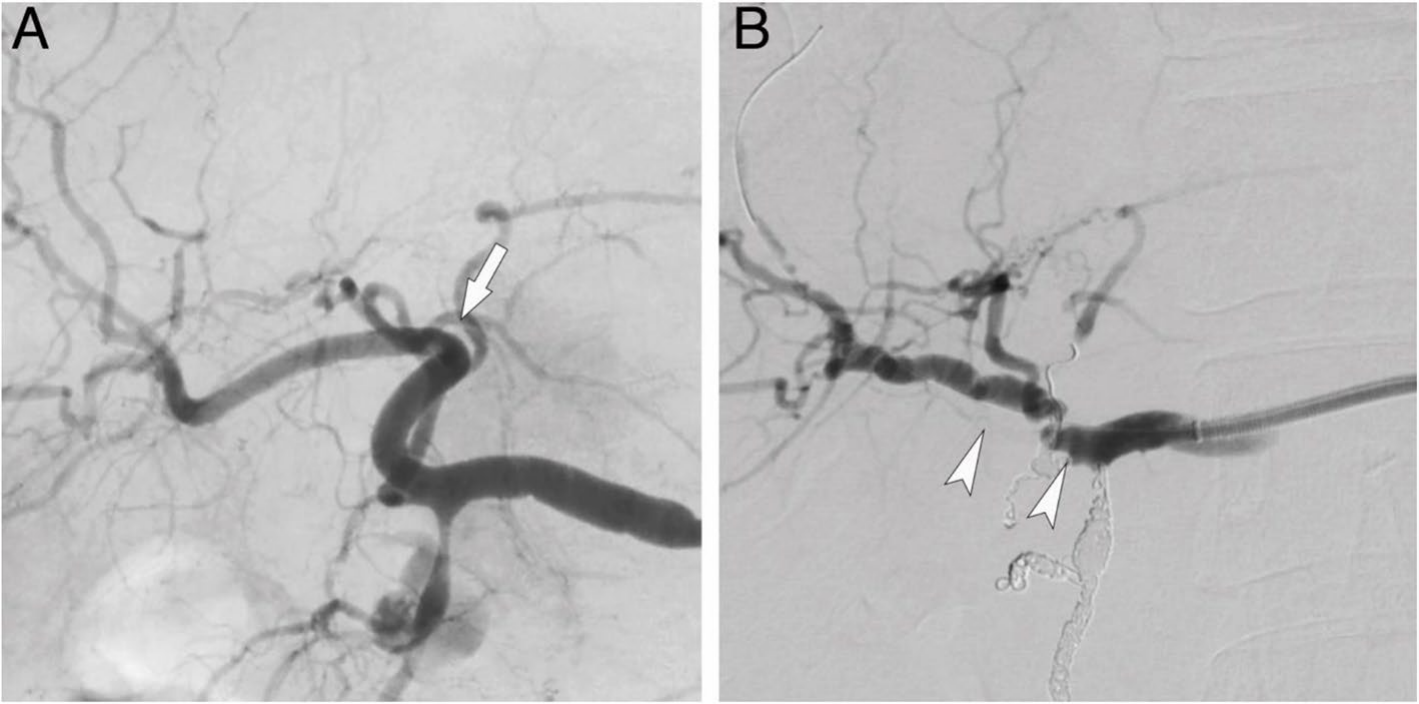
Digital Subtraction Angiography Before and After Vascular Linearization. This figure presents pre-procedural and post-procedural digital subtraction angiography (DSA) images. Arrowheads mark accordion-like appearances, while arrows show vessel tortuosity. **A** Sever tortuosity. **B** Severe tortuosity resulting in excessive accordion-like appearances and steps

For the qualitative evaluation, this an interventional radiologist with 13 years of experience and another with 20 years of experience independently reviewed the images and reached a consensus. Upon straightening the tortuous vessels with a stiff guidewire, three distinct morphological patterns were observed: vessels without an accordion-like appearance, vessels with an accordion-like appearance, and vessels with an accordion-like appearance accompanied by step-like deformities. Vessels without an accordion-like appearance straightened smoothly upon insertion of the stiff guidewire, exhibiting no morphological changes. Vessels with an accordion-like appearance displayed smooth, sinusoidal undulations upon straightening. These undulations were gentle and did not involve sharp angulations or abrupt changes in vessel direction. Vessels with an accordion-like appearance accompanied by step-like deformities not only exhibited the sinusoidal undulations but also developed additional step-like deformities or sharp angulations upon straightening (Figs. 2 and 3). These additional deformities suggest a higher degree of mechanical stress on the vessel wall.

#### Quantitative assessment of arterial diameter and vessel tortuosity before straightening

**Fig. 4:**
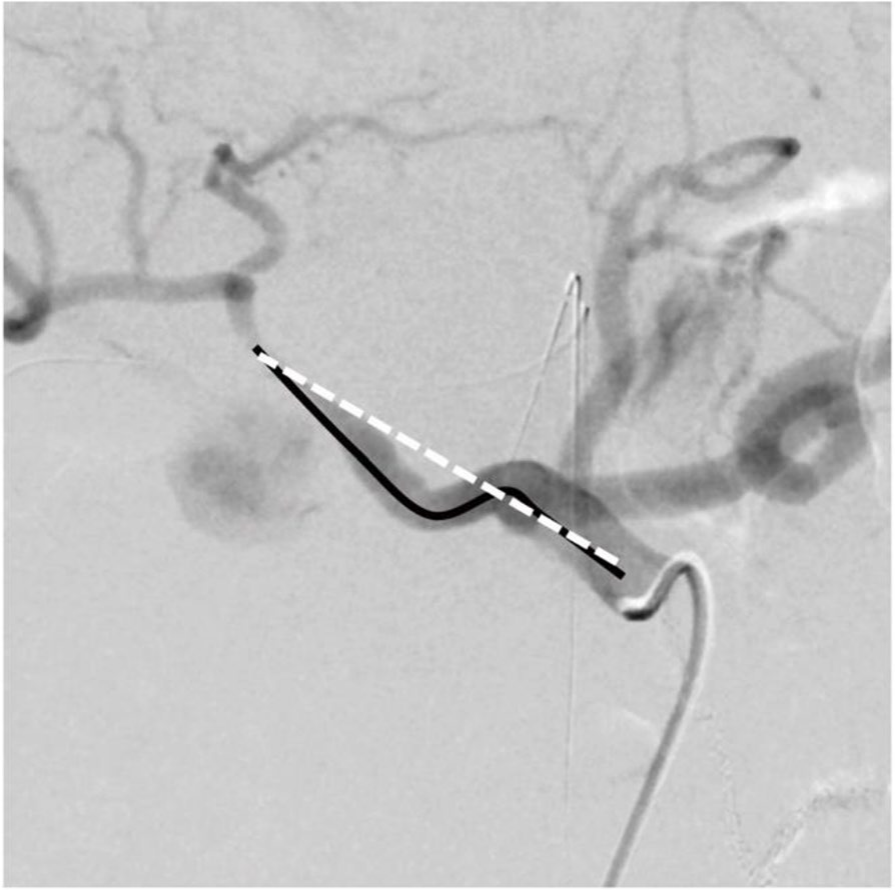
Quantification of Vessel Tortuosity. Digital subtraction angiography images illustrating the tortuosity quantification technique before vascular linearization. The ratio of the actual traced vessel path length to the straight-line distance from the coeliac axis to the vessel distal to the arterial injury is calculated. Black solid lines represent the actual vessel path, while white dashed lines indicate the straight-line reference, ensuring clear distinction in both color and black-and-white printed versions

The arterial diameter and vessel tortuosity were quantitatively assessed by an interventional radiologist with 13 years of experience. Vessel tortuosity was calculated by tracing the arterial path from the celiac artery to the bleeding point on DSA images and dividing it by the straight-line distance between these two points (Fig. 4). This ratio provided a quantitative measure of vessel tortuosity, allowing for objective comparison between cases. The degree of vessel tortuosity was quantified and plotted in all cases.

#### Qualitative assessment of vessel narrowing

**Fig. 5:**
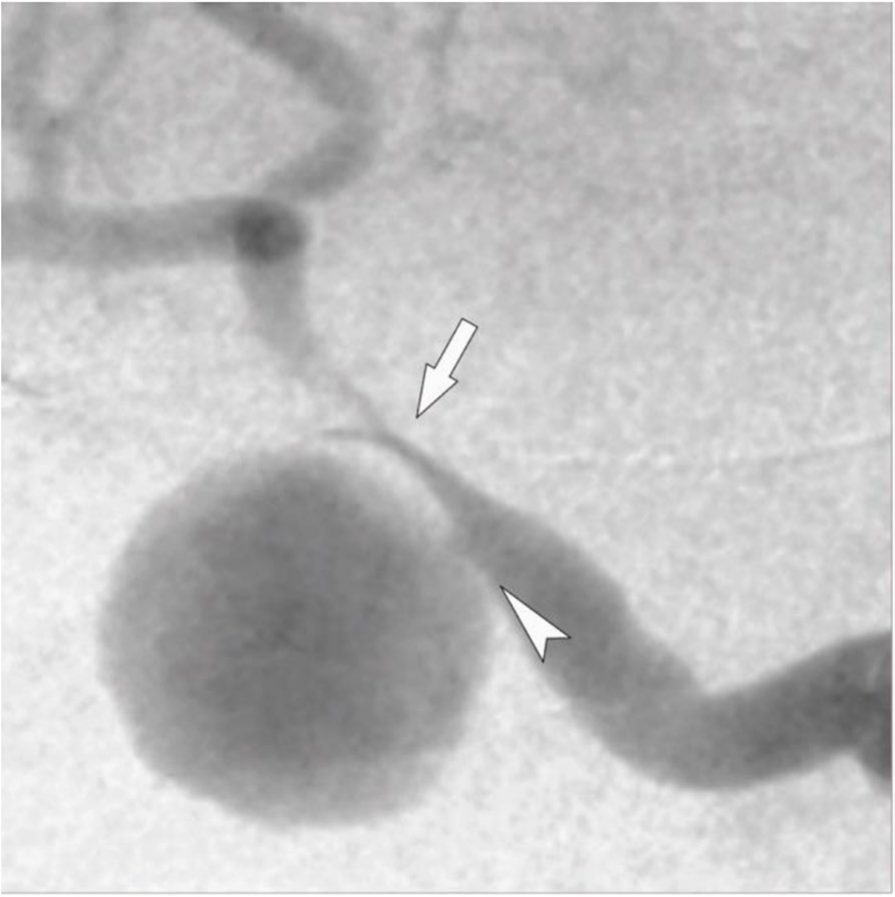
Angiographic Visualization of Arterial Injury Sites and Vessel Morphology. This figure presents digital subtraction angiography images showing bleeding sites and the morphology of vessels distal to arterial injuries and bleeding (AIB) points. Arrowheads indicate points of bleeding, while arrows highlight areas of vessel narrowing. Severe narrowing is observed distal to the AIB points

Vessel narrowing distal to the bleeding point was qualitatively classified into three categories: 'normal,' 'mild narrowing,' and 'severe narrowing' (Fig. 5). Normal indicates no noticeable constriction. Mild narrowing was defined as a noticeable constriction that is more pronounced than normal but does not have a thread-like appearance. Severe narrowing was characterized by a significant constriction with a thread-like appearance.

### Statistical analysis

We used Fisher's Exact Test and the Mann–Whitney U test for all statistical analyses. Fisher's Exact Test, ideal for small sample sizes, was used to assess the relationships between categorical variables, including the use of a stiff wire and the development of steps in accordion-like vessel morphology, vessel narrowing distal to the bleeding point and stent occlusion, severe narrowing and overlapping stent extension, and overlapping stent extension and stent occlusion. The Mann–Whitney U test compared means between independent samples without assuming a normal distribution, analyzing the impact of vessel morphology on the time to stent placement. Statistical significance was set at *p* < 0.05. Quantitative values of vessel tortuosity were analyzed by creating dot plots and comparing vessel straightening and serpentine features with procedural variations. The relationship between this period and the degree of vessel narrowing with stent occlusion was also examined.

## Results

### Stent placement

Table 1 presents the demographic and clinical data of the 11 patients included in this study (eight men, three women; median age = 64.5 years, range 48–80). We treated 12 consecutive cases involving vascular extravasation or suspected pseudoaneurysms due to postoperative vascular injury that required stent placement.

**Table 1:** Patient characteristics and viabahn stentgraft placement procedure

Case No	Age	Sex	UD	SP	TIFS (d)	SB	VTI	AD (mm)	VN	SGD (mm)	Total SGL (mm)	OL (mm)	SBE	ITS^a^	FTS	CS	Patency (One month)
1	54	M	EC	PPPD	25	GDA stump	1.37	5.9	N	7	2.5	0	LHA	N	Y	Y	Y
2	69	M	DPC	PD	25	GDA stump	1.28	5.1	S	5/6	3.5	1.5	LHA	Y	Y	Y	N
3	48	F	PSC	LDLT	42	PHA	1.04	4.3	N	5/6	3	2	N.A	Y	Y	Y	Y
4	49	M	P-NET	DP	14	SA stump	1.05	10.7	N	13	5	0	LGA	Y	Y	Y	Y
5	78	F	EC	SSPPD	22	GDA stump	1.02	3.8	M	5	2.5	0	N.A	Y	Y	Y	Y
6	79	M	EC	SSPPD	29	CHA	1.20	3.6	S	5	3.5	1.5	N.A	Y	Y	Y	N
7	58	M	PC	PD	6	RHA	1.04	4.5	N	5	3.5	1.5	N.A	Y	Y	Y	Y
8	80	M	EC	PD	19	GDA stump	1.11	4.4	M	5	2.5	0	N.A	Y	Y	Y	Y
9	55	M	PC	PD	22	GDA stump	1.14	5.7	S	6/7	3.5	1.5	N.A	Y	Y	Y	Y
10	76	M	HC	LR	14	RHA	1.41	4.3	N	5/6	3.5	1.5	N.A >	N	Y	Y	Y
11	76	M	HC	LR	31	LHA stump	1.29	4.5	N	6	5	2.5	N.A	N	Y	Y	Y
12	60	F	PC	DP	21	GDA	1.06	4.6	N	6	2.5	0	GDA/LHA/SDA	Y	Y	Y	Y

*UD* under disease, *SP* surgical procedure, *TIFS* time interval from surgery, *SB* site of bleeding, *VTI* vessel tortuosity index, *AD* arterial diameter, *VN* vessel narrowing, *S* severe narrowing, *M* mild narrowing, *N* None, *SGD* stentgraft diameter, *SGL* stentgraft length, *OL* overlap length, *SBE* sidebranch embolization, *ITS* initial technical success, *FTS* final technical success, *CS* clinical success, *Patency* stentgraft patency, *EC* extrahepatic cholangiocarcinoma, *DPC* duodenal papillary carcinoma, *PSC* primary sclerosing cholangitis, *P-NET* pancreatic neuroendocrine tumors, *HC* hilar cholangiocarcinoma, *PC* pancreatic cancer, *PPPD* pylorus preserving pancreatoduodenectomy, *PD* pancreaticoduodenectomy, *LDLT* living donor liver transplantation, *DP* distal pancreatectomy, *SSPPD* subtotal stomach-preserving pancreaticoduodenectomy, *LR* liver resection, *GDA* gastroduodenal artery, *PHA* proper hepatic artery, *SA* superior artery, *CHA* common hepatic artery, *RHA* right hepatic artery, *LHA* left hepatic artery, *LGA* left gastroepiploic artery, *SDA* superior duodenal artery. In the columns "Technical Success," "Clinical Success," and "1-Month Stent Graft Patency," "Y" denotes Yes and "N" denotes No

^a^The initial technical success refers to whether the stent insertion attempt using a stiff wire was successful

The injuries observed included vascular extravasation or suspected pseudoaneurysms, with 58% (7/12) located at vascular stumps and 42% (5/12) occurring within the main vessels. Vessel narrowing distal to the bleeding point was classified as severe in 25% (3/12) and mild in 17% (2/12) of cases.

In 75% (9/12) of cases, we used stiff guide wires to deploy the stents successfully, regardless of the presence of an accordion-like appearance upon straightening. However, in 25% (3/12) of cases with high tortuosity quantification values, straightening led to accordion-like appearances and steps, making it challenging for the stent to advance over the stiff wire. In these cases, we had to stop the procedure and switch to softer guide wires, along with other adjustments, to facilitate successful stent deployment. The relationship between accordion-like appearances and the technique's success using a stiff wire was statistically significant (*p* = 0.005) (Table 2).

**Table 2:** Associations among vessel morphology, stent length management, procedural success using stiff wire, and stent occlusion within one month after placement

	**Initial stent insertion using a stiff wire**		
	**Success** **n = 9**	**Failure** **n = 3**	***P* value**
**Vessel morphology in straightening**			
**No change/accordion-like without steps**	9 (100)	0 (0)	0.005
**Accordion-like with steps**	0 (0)	3 (100)	
	**Stent patency after one month**		
	**Yes** **n = 10**	**No** **n = 2**	
**Vessel distal to the bleeding point**			
**No/Mild narrowing**	9 (90)	0 (0)	0.045
**Severe narrowing**	1 (10)	2 (100)	
**Extension of the total stent length**			
**Single stent placement**	5 (50)	0 (0)	0.47
**Extension by overlapping stents**	5 (50)	2 (100)	

This table elucidates the interrelationships among vessel morphology, stent length management strategies, the use of stiff guidewires during procedures, and the incidence of stent occlusion within one month following placement. It comprises three separate 2 × 2 analyses: Procedural Success Using Stiff Wires versus Vessel Morphology; Pre-Placement Vessel Narrowing versus Stent Patency at One Month; and Stent Graft Extension through Overlapping Techniques versus Stent Patency at One Month. Statistical significance for each association was determined using Fisher's Exact Test, with p-values below 0.05 considered indicative of significant relationships

We used Viabahn stent-grafts with the following diameters: 13 mm (8%, 1/12), 5 mm (41%, 5/12), 6 mm (17%, 2/12), a combination of 5 mm and 6 mm (17%, 2/12), 6 mm and 7 mm (8%, 1/12), and 7 mm (8%, 1/12). In terms of stent length, a single 2.5 cm stent was placed in 33% (4/12) of cases, and a single 5 cm stent in 8% (1/12) of cases. Overlapping stent grafts to extend the placement length were used in 58% (7/12) of cases. Specifically, in six cases where obtaining a sufficient landing zone of at least 1 cm was not feasible due to the extent or location of the vascular injury, we decided to extend the stent graft placement to ensure complete coverage and secure anchoring of the stent. Additionally, in one case, vessel straightening during stent deployment led to the formation of steps and subsequent blood flow reduction. To alleviate this issue and restore adequate blood flow, we extended the stent to smooth out the vessel contour. Side branch embolization was performed in 4 out of 12 cases (33%).

The initial technical success rate using a stiff wire was 75% (9/12). After making necessary adjustments, such as switching to a softer wire, the overall technical success rate reached 100% (12/12). Clinical success rates were also 100% (12/12). Stent patency one month post-operation was 83% (10/12). In one patient, after treating the first rupture, an additional injury was identified, requiring a second stent graft treatment (Table 1).

### Impact of vessel narrowing, stent extension by overlapping, and placement timing on stent occlusion

**Fig. 6:**
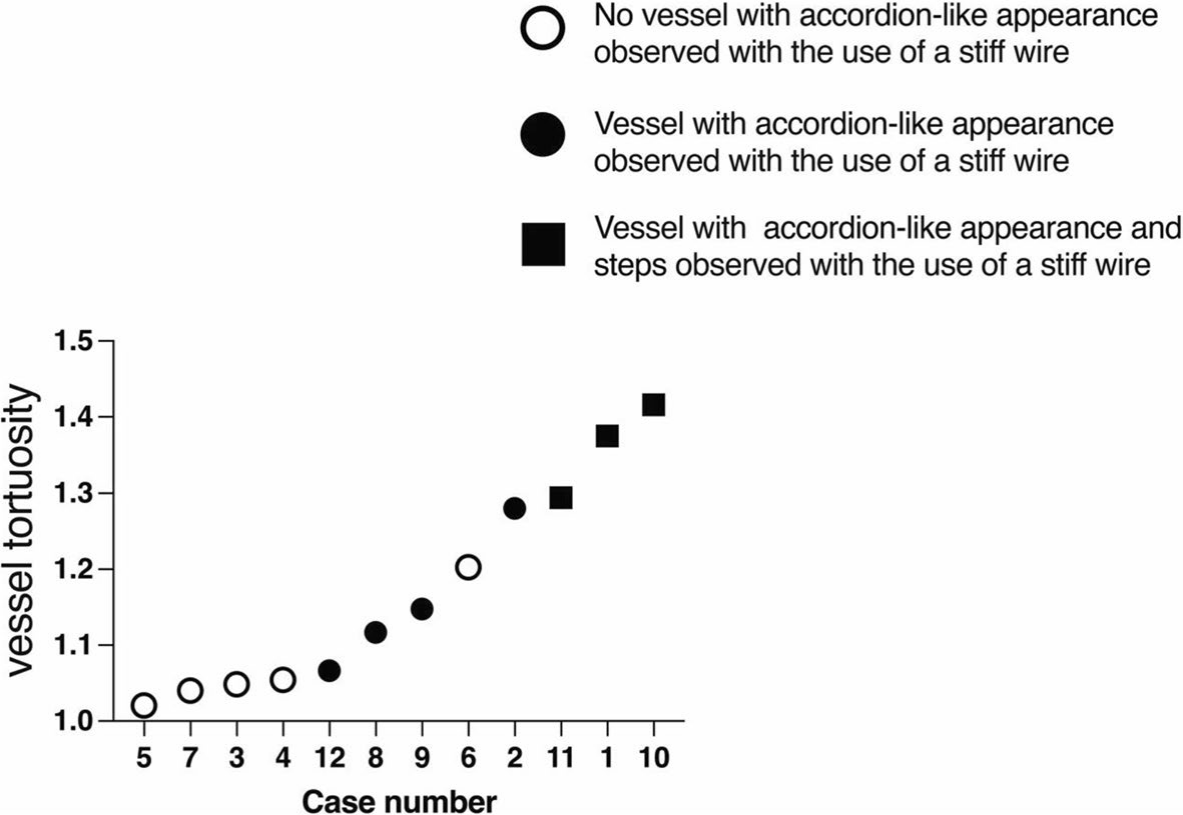
Scatter Plot Displaying the Value of Vessel Tortuosity for Each Patient Case. Black circles represent cases where accordion-like vascular patterns emerged due to vessel straightening, while white circles denote cases without such patterns. Black squares indicate cases where both accordion-like vessel patterns and step formations occurred, necessitating alternative techniques, such as switching to a softer guidewire, among other adjustments

A typical pattern of narrowing distal to the AIB point at stent placement is shown in Fig. 6. Among cases with severe narrowing, 67% (2/3) experienced occlusion within one month, a statistically significant difference (*p* = 0.045) (Table 2). Among the cases where the stent became occluded within one month, all cases involved extending the stent by overlapping nearly half of its original stent length; however, there was no statistically significant difference (*p* = 0.47) (Table 2). Additionally, there was no statistically significant association between severe narrowing and the extension of the stent by overlapping (*p* = 0.20) (data not shown).

### Complications and mortality

Furthermore, no complications, including ischemia, requiring treatment or deaths occurred within one month in any of the cases, underscoring the safety profile of the Viabahn stent graft in our study cohort.

## Discussion

This study investigated the association between vessel morphology and the technical success of stent placement, as well as short-term occlusion at one month post-operation. In cases of severe tortuosity, the formation of steps during vessel straightening made stent insertion challenging, necessitating specific countermeasures (*p* = 0.005). Vessel narrowing distal to the arterial injuries and bleeding (AIB) point, when presenting as thread-like narrowing, was significantly associated with higher rates of short-term occlusion at one month (*p* = 0.045).

The Viabahn stent graft has shown effectiveness and safety for visceral AIB, with technical success rates ranging from 67–96% [21–23, 29]. Our study observed a final technical success rate of 100%, aligning with these prior published series. However, these studies have not clarified the factors determining success rates, which are likely influenced by patient selection and the type of stent graft used. Advancing the guiding sheath beyond the damaged area during stent graft delivery may enhance the procedure’s success rate, as demonstrated in previous studies. Additionally, the use of stiff supportive guidewires has been reported to increase success rates [30, 31].

In our study, we used stiff guide wires in 75% (9/12) of cases to deploy stents successfully, regardless of the presence of an accordion-like appearance upon straightening. However, in 25% (3/12) of cases with high tortuosity quantification values, straightening led to accordion-like appearances and steps, making it challenging for the stent to advance over the stiff wire. In these instances, we had to switch to softer guide wires and make other adjustments to facilitate successful stent deployment. The relationship between accordion-like appearances and the technique's success using a stiff wire was statistically significant (*p* = 0.005) (Table 2), highlighting the impact of vessel tortuosity on technical outcomes.

The clinical success of stent grafts at one month remains high, with patency rates reported between 81–100% [19, 32–34] and patency rate of stent grafts decreases over time [21, 23, 30]. Risk factors for stent graft occlusion include placement in vessels < 4 mm or oversizing the stent graft by > 20% [22]. Consistent with these findings, our study observed a 1-month patency rate of 83%, which aligns with previously reported rates. And among the three cases exhibiting severe narrowing distal to the AIB point, two experienced occlusion within one month. These occlusions occurred in stents placed in the common hepatic artery and the gastroduodenal artery stump, suggesting that distal vessel narrowing was likely caused by external compression, such as from a hematoma. This distal narrowing appeared to be related to the stent occlusion, possibly due to the development of collateral pathways, which may have progressively reduced blood flow within the stent. The small sample size of our study may have limited the statistical power to detect this association, indicating that these findings should be interpreted with caution. Further studies with larger cohorts are needed to investigate this potential relationship and identify patients at higher risk of occlusion.

Additionally, both cases that occluded within one month involved the extension of the stent graft by overlapping additional stents. Extension of the stent graft has been suggested as a risk factor for early occlusion in the iliac artery region [35]. Moreover, overlapping stents are known to significantly increase axial stiffness [36]. From a fluid dynamics perspective, the placement of stents may cause localized turbulent blood flow due to vortices, potentially increasing the risk of occlusion [37]. These insights suggest that the stent lumen itself might be a site for blood flow disturbances, and the increased axial stiffness due to the stents could lead to deformations in the vessel both proximal and distal to the stent post-placement, further contributing to hepatic artery flow impairments. Nonetheless, the clinical impact of these occlusions might be minimal, allowing time for collateral pathways to develop and maintain adequate blood flow. Nonetheless, the clinical impact of these occlusions might be minimal, allowing time for collateral pathways to develop and maintain adequate blood flow.

This study has several limitations. First, it is a retrospective, single-institution analysis with a small sample size, which may limit our ability to detect statistically significant associations between vessel narrowing and occlusion rates. Consequently, clinically relevant trends might not have reached statistical significance, underscoring the need for larger studies to validate our findings. Second**,** the index of tortuosity was only assessed on two-dimensional angiograms, which may lead to an underestimation of vessel tortuosity. Additionally, the qualitative evaluation of stenosis and the presence or absence of steps in the hepatic artery is prone to inter-observer variability.

## Conclusions

In conclusion, during Viabahn stent graft placement, vessel tortuosity can cause an accordion-like appearance when the vessel is straightened, which may create steps and complicate stent insertion. Although stent placement is feasible in severe narrowing distal to the arterial injuries and bleeding point, it significantly increases the risk of short-term occlusion.

## Data Availability

The datasets used and/or analysed during the current study are available from the corresponding author on reasonable request.

## Abbreviations

AIB: Arterial injuries and bleeding
DSA: Digital subtraction angiography
